# Characterization of capsule genes in non-pathogenic *Neisseria* species

**DOI:** 10.1099/mgen.0.000208

**Published:** 2018-08-03

**Authors:** Marianne Elizabeth Alexandra Clemence, Martin Christopher James Maiden, Odile Barbara Harrison

**Affiliations:** Department of Zoology, University of Oxford, Oxford, UK

**Keywords:** meningitis, colonization, virulence determinants, transmission, invasive

## Abstract

The genus *Neisseria* comprises a diverse group of commensal bacteria, which typically colonize the mucosal surfaces of humans and other animals. *Neisseria meningitidis*, the meningococcus, is notable for its potential to cause invasive meningococcal disease (IMD) in humans; however, IMD is comparatively rare, and meningococci normally colonize the nasopharynx asymptomatically. Possession of a polysaccharide capsule has been shown to be a prerequisite for disease in almost all IMD cases, and was previously considered unique to *N. meningitidis*, and potentially acquired by horizontal genetic transfer (HGT). Nevertheless, the capsule must also have some role in asymptomatic colonization and/or transmission, consistent with the existence of six non-disease-associated meningococcal capsule serogroups. In this study, full complements of putative capsule genes were identified in non-pathogenic *Neisseria* species, including *Neisseria subflava* and *Neisseria elongata*. These species contained genes for capsule transport and translocation homologous to those of *N. meningitidis*, as well as novel putative capsule synthesis genes. Phylogenetic analyses were consistent with the proposal that these genes were acquired by the meningococcus through HGT. In contrast with previous evolutionary models, however, the most parsimonious explanation of these data was that capsule transport genes had been lost in the common ancestor of the meningococcus, gonococcus, and their close relatives, and then reacquired by some meningococci. The most likely donor of the meningococcal transport genes was another *Neisseria* species.

## Data Summary

This work made use of sequencing data obtained from the pubMLST database (https://pubmlst.org/neisseria), the NCTC 3000 project (https://www.sanger.ac.uk/resources/downloads/bacteria/nctc/) and GenBank (https://www.ncbi.nlm.nih.gov/genbank/index.html). A comprehensive list of strain IDs, metadata and accession numbers can be found on FigShare at https://doi.org/10.6084/m9.figshare.6016112.v1. Annotated pubMLST isolates can be searched in the pubMLST isolate database, and allele sequences retrieved using the NEIS numbers from the pubMLST sequence database.

**Impact Statement**

*Neisseria meningitidis*, whilst normally a harmless commensal of the human nasopharynx, can cause invasive meningococcal disease (IMD), comprising meningitis and/or septicaemia. Expression of a polysaccharide capsule is essential for IMD, but must also be involved in asymptomatic colonization. The capsule has been considered a virulence factor unique to *N. meningitidis*; however, here a full complement of homologous putative capsule genes was identified in non-pathogenic *Neisseria* (NPN) species. NPN species are important members of the human nasopharyngeal microbiota, as well as coexisting with the meningococcus in the nasopharynx. The results inform debate about the acquisition of capsule by the meningococcus, an important step in the emergence of pathogenic potential.

## Introduction

The genus *Neisseria* is a diverse group of Gram-negative bacteria, many of which are asymptomatic colonizers of the mucosal surfaces of animals and man [1]. In humans, they have been isolated from the mouth, nose, throat and urogenital tract, but whilst many *Neisseria* species belong to the human oral microbiota, research has focused on those associated with disease: *Neisseria gonorrhoeae* and *Neisseria meningitidis*. In common with many other *Neisseria* species, *N. meningitidis* usually colonizes the nasopharynx asymptomatically; however, it occasionally invades the bloodstream, leading to life-threatening invasive meningococcal disease (IMD), comprising meningitis and/or septicaemia [2]. In contrast, there are very few case reports of other *Neisseria* species causing invasive disease. Compared to asymptomatic colonization, IMD is an extremely rare transmission-terminating event, associated with particular meningococcal genotypes that normally express a polysaccharide capsule [3].

Capsules are associated with virulence in several human pathogens, including *Escherichia coli*, *Haemophilus influenzae* and *Klebsiella pneumoniae* [4–6]. A number of successful vaccines have been developed that target capsular antigens, for example the polysaccharides forming the capsules of the meningococcal serogroups A, C, W and Y [7]. Capsules can aid evasion of immune responses, including the complement system and phagocytosis by macrophages, facilitating persistence in the bloodstream [8, 9]; however, capsules have also been identified in free-living bacteria and symbionts [10, 11]. In both *N. meningitidis* and other species, association with disease is often restricted to a subset of capsular groups or types [2, 5, 6], indicative that in general the capsule confers benefits during transmission or protects the bacteria from local inflammation in the nasopharynx [12, 13].

In *N. meningitidis,* the capsule is produced via ABC transporter-dependent polymerization, whereby synthesis and polymerization of the polysaccharide take place at the bacterial inner membrane, prior to transport across the membrane and translocation to the cell surface [14]. These processes are encoded by genes located in the *cps* locus [15], which is functionally divided into several contiguous regions. Region A contains genes involved in capsule synthesis, in particular glycosyltransferases and capsule polymerases, but also other proteins involved in additional capsule modifications, and sometimes insertion sequences are present [15, 16]. This region is highly variable, with 12 known variants corresponding to the 12 meningococcal serogroups. Of these, only six (A, B, C, W, X and Y) are associated with disease. Regions B and C are composed of the genes *ctrEF* and *ctrABCD*, respectively, and are required for capsule translocation and transport. These regions are well conserved throughout *N. meningitidis*, unlike region A [15]. Region D of the *cps* contains the genes *rfbABC* and *galE*, which are thought to play a role in LPS synthesis [17]. A duplication of region D, containing a truncated *galE,* is designated region D’ [18]. Finally, region E contains the gene *tex* and two pseudo cytosine methyltransferases of unknown function. Although regions D, D’ and E are not directly involved in capsule synthesis or transport, they are generally considered part of *cps* due to their location within the locus. Isolates that do not contain regions A, B and C are described as capsule null, and instead possess a distinct 113–118 bp sequence located between regions D and E, the capsule null locus (*cnl*) [19]. The *cnl* locus has also been identified in *N. gonorrhoeae* and the non-pathogenic *Neisseria* (NPN) species *Neisseria lactamica* [19], and no encapsulated isolates from these species have been described.

Whilst a number of putative virulence genes have been found in NPN species [20], the capsule has been considered to be unique to the meningococcus [21], possibly acquired in a horizontal genetic transfer (HGT) event that gave rise to the potentially pathogenic variants of *N. meningitidis* [3, 22]. In this study, capsule genes have been identified and characterized in NPN species, which typically do not cause disease. These results have implications for understanding the acquisition of capsule in *N. meningitidis*.

## Methods

### Isolate collection and species definitions

Whole-genome sequence (WGS) data from *Neisseria* isolates were obtained from pubmlst.org/neisseria, which is hosted on the Bacterial Isolate Genome Sequence Database (BIGSdb) genomics platform [23]. At the time of writing, the database contained WGS data from >13 000 *Neisseria* isolates, 235 of which were from NPN species. The pubMLST sequence database contains defined *Neisseria* loci and allele sequences, with each locus assigned a unique NEIS number. Isolates can be annotated with NEIS loci automatically or manually through a blast-based process, and new alleles are assigned an arbitrary allele number. Most of the WGSs in pubMLST are high-quality draft genomes, with sequencing reads assembled into approximately 100–300 individual contigs.

A total of 20 species had been defined in the pubmlst.org/neisseria database using the universal and high-resolution ribosomal multi-locus sequence typing (rMLST) approach [24]. These species included the human nasopharyngeal commensals *Neisseria polysaccharea* (16 isolates), ‘*Neisseria bergeri’* (1 isolate), *N. lactamica* (140 isolates), *Neisseria cinerea* (15 isolates), *Neisseria subflava* (19 isolates), *Neisseria oralis* (4 isolates), *Neisseria mucosa* (10 isolates), *Neisseria elongata* subsp. (4 isolates) and *Neisseria bacilliformis* (4 isolates), and the pathogens *N. meningitidis* and *N. gonorrhoeae*, along with several species isolated from animals or animal-bite wounds. *N. meningitidis* region A genes were sourced from isolate sequences for 0106/93 and 0084/93 published in pubMLST, and 29 043 published in GenBank (accession no. HF562984.1).

All NPN isolates in pubMLST were surveyed for the presence of *cps* genes that putatively encoded a polysaccharide capsule. All isolates found to contain a *cps* were further characterized. Isolates from additional species were included in phylogenetic analyses. Capsule sequence data from other genera were obtained from GenBank. Reference genomes were obtained from either GenBank (http://www.ncbi.nlm.nih.gov/genbank/index.html) or the NCTC 3000 project (http://www.sanger.ac.uk/resources/downloads/bacteria/nctc/).

### Annotation of *cps* in NPN isolates

The BIGSdb software enables blast searches of protein or nucleotide sequences against genomes contained within pubMLST. Region B and C *cps* genes *ctrABCDEF*, which are involved in capsule transport in the meningococcus, had previously been defined in the pubMLST.org/neisseria sequence database as NEIS0055, NEIS0056, NEIS0057, NEIS0058, NEIS0066 and NEIS0067, respectively. For each gene, the amino acid sequence of allele 1 was used as a pBLAST query against all available NPN isolates within the database for which WGSs were available. Candidate genes were annotated in Artemis [25] and G+C content determined in mega 7 [26]. The same approach was also used to annotate region D and E genes, and any other relevant genes, where necessary. Annotations were uploaded as novel alleles in pubMLST.

The proposed *cps* regions of NPN isolates were further annotated in Artemis [25]. ORFs adjacent to proposed region C genes were queried against the National Center for Biotechnology Information (NCBI) RefSeq protein database using pBLAST and the Pfam database [27], as well as the pubMLST sequence database. Support for putative region A genes was based on homology to capsule synthesis genes from *N. meningitidis* and/or other bacterial species, or at least for gene products consistent with a function in capsule synthesis, such as glycosyltransferases. Additional guidance was based on comparisons of synteny with *N. meningitidis* and between NPN isolates and species. In this way, ORFs that did not contain significant homology to previously described capsule synthesis genes, or previously described capsule synthesis-like genes, could only be included in a putative region A if they were flanked by ORFs that did have significant homology. Region A candidates were also queried against the non-redundant sequences of the CAZy database [28], which contains data on carbohydrate-active enzyme families, using the CAZymes Analysis Toolkit [29]. CAZy families were predicted using both sequence similarity and Pfam rule-based annotations, with an *E* value threshold of 1×10^−10^ and bit score threshold of 55. Annotations were uploaded as novel NEIS loci and alleles in pubMLST. The organization of the *cps* found in NPN isolates was compared with that of the meningococcus and visualized using genoPlotR [30].

### Identification of homologous candidate region A genes

Potentially homologous genes shared by isolates from the same species were identified based on gene order, sequence length and predicted function. The nucleotide sequences for each gene were aligned in Clustal Omega [31], and paired identity matrices (PIMs) were generated using Clustal 2.1. Suspected homology between different species was identified in the same way and investigated using pairwise comparisons of amino acid sequences generated by Clustal, and pairwise tBLASTx comparisons between the proposed region A of each species were made using the Artemis Comparison Tool [32] and visualized using genoPlotR.

### Phylogenetic analyses

A recombination-corrected phylogenetic tree of *Neisseria* isolates, with *Moraxella catarrhalis* as an outgroup, was generated based on rMLST loci [24]. The nucleotide sequences of 51 of the 53 genes that constitute the protein subunits of the ribosome (excluding *rpmE* and *rpmJ*, as they are paralogous in some *Neisseria*), were extracted from each isolate using the BIGSdb genome comparator, and aligned using mafft [33]. A maximum-likelihood (ML) tree was generated in PhyML v3.1 [34] using the GTR+I+G substitution model, determined to be the best-fit model by jModelTest v2.1.10 [35], with 100 bootstrap replicates. The tree was corrected for recombination in ClonalFrameML [36], and rendered and annotated using the ete 3 toolkit [37]. The phylogeny included all isolates belonging to those species in which capsule genes were identified, and representatives of *N. cinerea, N. lactamica, ‘N. bergeri’, N. polysaccharea* and *N. gonorrhoeae,* in none of which were capsule genes identified, and representatives of *N. meningitidis*.

Where present, region B and C genes were also extracted from isolates in the dataset. Additionally, homologous capsule genes, as determined by NCBI blast queries, were extracted from isolates of *Mannheimia haemolytica*, *Actinobacillus pleuropneumoniae*, *Actinobacillus suis*, *Bibersteinia trehalosi*, *H. influenzae*, *Pasteurella multocida* and *Kingella kingae*. Amino acid sequences from all species were concatenated, aligned with muscle [38] and trimmed in trimAL v1.2 [39] to remove columns with gaps in more than 20 % of sequences or with a similarity score lower than 0.001. An unrooted ML tree was generated in PhyML using the LG+I+G substitution model, selected to be the best fit by ProtTest v3.4.2 [40]. The tree was rendered in FigTree v1.4.3 (http://tree.bio.ed.ac.uk/software/figtree/).

## Results

### Identification of *cps* in NPN species

**Fig. 1.**
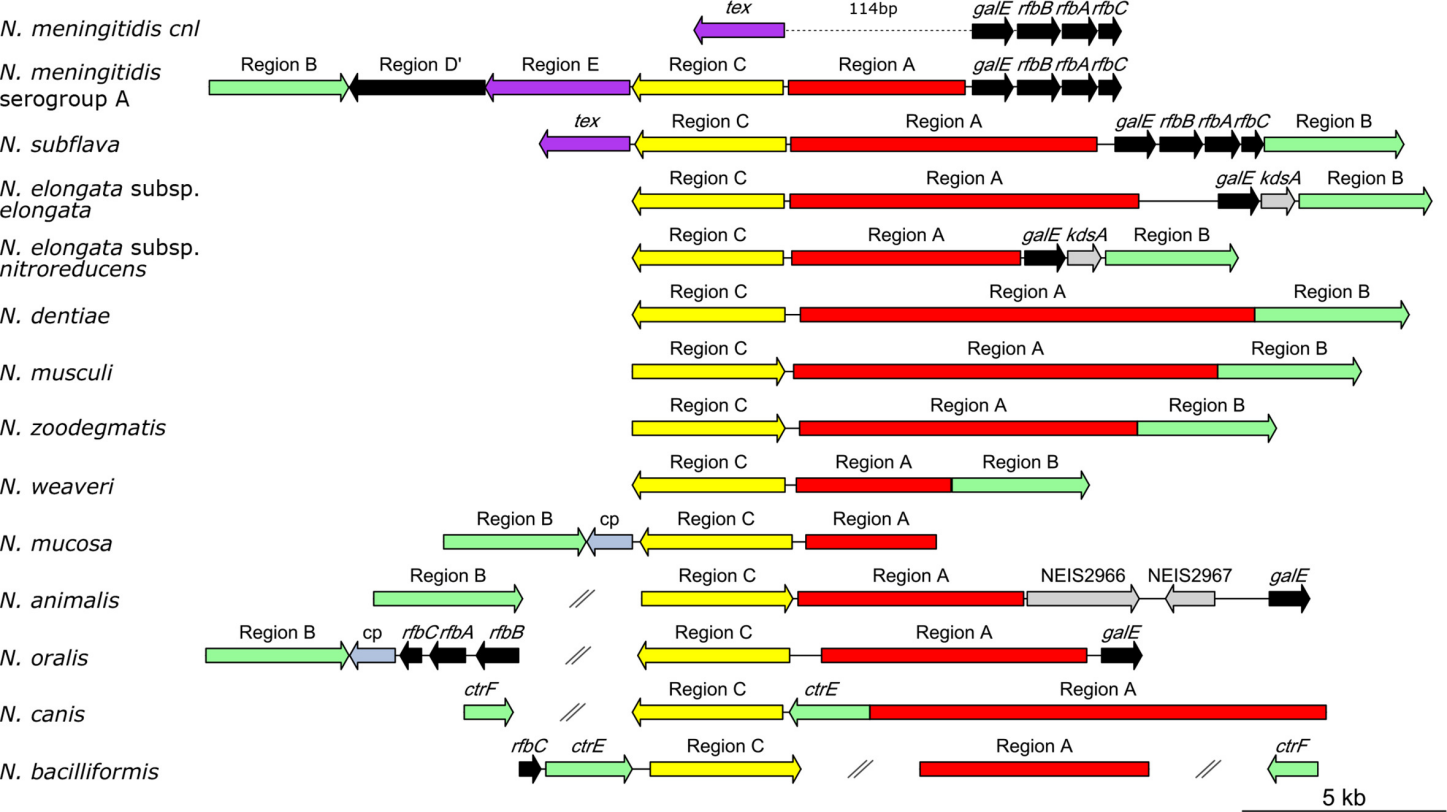
Arrangement of *cps* across the genome in *Neisseria* species. Region A is involved in capsule synthesis, region B in capsule transport and region C in capsule translocation. *N. animaloris* and *N. wadsworthii* were excluded, since region A was interrupted in the genome assembly for the isolates from these species. Two diagonal lines represent >5 kb between genes. cp refers to capsule phosphotransferase, as seen in some W, I and K isolates of *N. meningitidis* [15].

Capsule gene homologues were identified in isolates from a total of 13 *Neisseria* species contained within the pubmlst.org/neisseria database [23], including *N. bacilliformis* (4 of 4isolates); *N. elongata* subsp. (2 of 4 isolates); *Neisseria musculi* (1 isolate); *Neisseria dentiae* (1 isolate); *Neisseria animaloris* (1 isolate); *Neisseria zoodegmatis* (1 isolate); *Neisseria weaveri* (1 isolate); *Neisseria canis* (1 isolate); *Neisseria wadsworthii* (1 isolate); *Neisseria animalis* (1 isolate); *N. oralis* (4 of 4isolates); *N. mucosa* (1 of 9 isolates); and *N. subflava* (17 of 19 isolates). Regions A, B and C were annotated in isolates of all of these species (Fig. 1), with the exception of *N. wadsworthii* and *N. animaloris*, in which region A was interrupted in the genome assembly.

With the exception of 11 *N. subflava* isolates, isolates in which capsule genes were identified were found to contain homologues for all six of the *N. meningitidis* region B and C genes (Table S1, available in the online version of this article), sharing 52–99 % amino acid sequence identity with the relevant query. Alignment length covered at least 92 % of the relevant query, except the homologues of *ctrF* in CCUG 50858 (85 %) and NJ9703 (71 %). This reduced query coverage was due to an incomplete gene located at the end of a contig in WGS data for isolate CCUG 50858, and a frameshift mutation in the sequences for isolate NJ9703.

Annotation using Artemis [25] showed that *ctrABCD* genes of region C were contiguous and in the same order as those found in *N. meningitidis*. The *ctrE* and *ctrF* genes were contiguous in all isolates except those from *N. canis* and *N. bacilliformis*, although in these two species *ctrE* was adjacent to region C. The remaining 11 *N. subflava* isolates were found to contain homologues for one or both of *ctrE* and *ctrF*, but no region C genes were identified (Table S1).

### Annotation of putative novel region A genes in NPN species

With the exception of *N. bacilliformis*, isolates that contained a complete set of region B and C genes possessed a region adjacent to region C that had not been defined in the pubMLST.org/neisseria database at the time of analysis. A total of 59 ORFs were annotated as putative region A genes. Of these, 33 were homologous, with capsule genes described in both *N. meningitidis* and in other non-*Neisseria* species, including *A. pleuropneumoniae* and *Mannheimia haemolytica*, and a further 16 were homologous with genes commonly involved in capsule synthesis, including glycosyltransferases and acetyltransferases (Table 1). Remaining genes were included as part of a putative region A based on synteny. In many cases, genes had been annotated previously in the relevant species in RefSeq, albeit not directly attributed to a NPN *cps*, and so blast hits were almost identical to the blast query. A total of 40 of the region A candidates belonged to a CAZy [28] glycosyltransferase family, based on either sequence similarity of Pfam rule-based annotation (Table 1). With the exception of GT61 (to which a gene in *N. weaveri* belonged), these were all glycosyltransferase families to which *N. meningitidis* region A genes belong.

**Table 1.** Highest pBLAST hits of candidate region A genes against RefSeq

**Species**	**NEIS locus**	**Product of highest non-hypothetical pBLAST hit/CAZy family**	**Identity (%)**	***E* value**	**G+C (mol%)**
*N. animalis* (CCUG 808)	2173	Glycerol-3-phosphate cytidylytransferase GT9*	99	0	43.6
	2947	Phosphotransferase	98	0	43.8
	2939	CDP-glycerol–glycerophosphate glycerophosphotransferase GT4	41	6^−31^	44.2
	2940	CDP-glycerol–glycerophosphate glycerophosphotransferase GT2	33	2^−37^	40.6
	2966	Galactosyl transferase GT32	33	3^−33^	41.1
	2967	α-/β-Hydrolase	31	5^−44^	45.8
*N. bacilliformis* (CCUG 38158)	2173	Glycerol-3-phosphate cytidylytransferase GT9*	100	1^−94^	37.6
	2938	Phosphotransferase	47	7^−118^	36.7
	2939	CDP-glycerol–glycerophosphate glycerophosphotransferase GT4	41	3^−24^	37.9
	2940	CDP-glycerol—glycerophosphate glycerophosphotransferase GT2	36	2^−36^	39.4
*N. canis* (CCUG 56775 T)	2157	UDP-*N*-acetylglucosamine 2-epimerase GT28*	74	0	40.3
	2737	UDP-*N*-acetyl-d-mannosamine dehydrogenase GT2*	87	0	43.4
	2738	Glycosyl transferase GT2	40	1^−92^	37.1
	2739	Glycosyl transferase GT4	45	0	38.9
	2740	Riboflavin synthase subunit β	28	4^−13^	31.3
	2968	α-/β-Hydrolase	34	3^−35^	30.2
	2741	*N*-Acetyltransferase	31	6^−40^	34.2
	2742	Spore coat protein GT4	81	0	38.5
*N. dentiae* (CCUG 53898)	2736	UDP-*N*-acetylglucosamine 2-epimerase GT28*	78	0	50.2
	2737	UDP-*N*-acetyl-d-mannosamine dehydrogenase GT2*	91	0	49.9
	2738	Glycosyl transferase GT2	59	6^−92^	35.4
	2739	Glycosyl transferase GT4	48	0	38.5
	2950	Asparagine synthase	22	1^−22^	31.6
	2741	*N*-Acetyltransferase	34	3^−37^	35.1
	2742	Spore coat protein GT4	90	0	46.4
*N. elongata* subsp. *elongata* (CCUG 2043T)	2965	Capsule biosynthesis protein GT4	100	0	31.4
	2941	Glycosyl transferase GT2	100	0	36.1
	2942	Acetyltransferase	100	0	37.9
	2943	α-/β-Hydrolase	38	1^−15^	34.6
	2974	d-Alanine–d-alanine ligase GT2	100	0	37.7
*N. elongata* subsp. *nitroreducens* (CCUG 30802T)	2173	Glycerol-3-phosphate cytidylytransferase GT9*	99	1^−89^	39.2
	2948	Phosphotransferase	51	1^−116^	33.7
	2939	CDP-glycerol–glycerophosphate glycerophosphotransferase GT4	31	1^−22^	36.5
	2940	CDP-glycerol–glycerophosphate glycerophosphotransferase GT2	35	9^−37^	37.4
*N. mucosa* (CCUG 431)	2972	Spore coat protein GT2	59	0	29.9
	2973	Acetyltransferase	45	8^−58^	33.5
*N. musculi* (AP2031)	2736	UDP-*N*-acetylglucosamine 2-epimerase GT28*	76	0	46.1
	2737	UDP-*N*-acetyl-d-mannosamine dehydrogenase GT2*	91	0	49.5
	2738	Glycosyl transferase GT2	38	4^−92^	39.6
	2739	Glycosyl transferase GT4	47	0	37.1
	2740	Riboflavin synthase subunit β	24	2^−10^	30.1
	2741	*N*-Acetyltransferase	35	6^−37^	40.8
	2742	Spore coat protein GT4	87	0	46
*N. oralis* (F0314)	2941	Glycosyl transferase GT2	100	0	39.6
	2943	α-/β-Hydrolase (variant in CCUG 804)	36	9^−14^	36.3
	2974	d-Alanine–d-alanine ligase GT2	100	0	37.98
*N. subflava* (NJ9703)	2184	Capsule biosynthesis protein GT4	100	0	43
	2941	Glycosyl transferase GT2 (variant in C102, C6A, CCUG 7826 and CCUG 24918)	100	0	39.7
	2942	Acetyltransferase (missing in C6A, CCUG 7826 and CCUG 24918)	100	0	37.2
	2943	α-/β-Hydrolase	38	6^−15^	36.1
	2974	d-Alanine–d-alanine ligase GT2	100	0	40.55
*N. weaveri* (CCUG 4007 T)	2944	Glycosyl transferase GT4	100	0	30.2
	2945	Capsule biosynthesis protein	99	0	27.5
	2946	Glycosyl transferase GT61*	29	2^−17^	28.6
*N. zoodegmatis* (NCTC 12230 T)	2736	UDP-*N*-acetylglucosamine 2-epimerase GT28*	77	0	40.7
	2737	UDP-*N*-acetyl-d-mannosamine dehydrogenase G2*	85	0	42.3
	2949	Glycosyl transferase GT4	34	3^−126^	37.6
	2971	Hypothetical	48	0	34.7
	2742	Spore coat protein GT4	81	0	37.8

*Indicates CAZy family predictions determined using Pfam rule-based annotations rather than sequence similarity.

In all but four species, the proposed region A was flanked on both sides by region B, region C, region D or some other gene with an unrelated function. In *N. animalis*, between the last putative capsule synthesis gene and *galE* was an ORF predicted to belong to the DUF1016 family; since this family is predicted to code for nuclease genes, it was considered unlikely to have a role in capsule synthesis. In *N. elongata* subsp. *elongata*, an IS*565* insertion sequence was identified between region A and *galE*, but no evidence was found to suggest that this was interrupting a putative capsule synthesis gene. In *N. canis*, the proposed region A was preceded by an IS*481* insertion sequence, but again no evidence was found showing that a putative capsule synthesis gene had been interrupted; ORFs adjacent to the insertion sequence had been previously annotated.

blast querying the *N. bacilliformis* isolates with all novel region A candidates identified putative region A genes homologous to those found in *N. elongata* subsp. *nitroreducens*. In *N. bacilliformis*, these four region A candidates were contiguous, but were located on different contigs from regions B and C. Region A candidates were not identified in *N. subflava* isolates that contained region B, but not region C. The G+C content of region A candidates was found to be lower than those typical for *Neisseria* genomes (49–54 %, but 60 % for *N. bacilliformis*) (Table 1). tBLASTx queries additionally identified homologues of meningococcal serogroup B/C/W/Y sialic acid synthesis genes *cssABC* in *N. weaveri* isolate CCUG 4007 T. These three genes were in a separate region of the genome of CCUG 4007 T and were distinct to the other candidate region A genes identified flanking regions B and C.

### Arrangement of *cps* in *N. meningitidis* and NPN species

None of the NPN *cps* were syntenic with the gene order seen in *N. meningitidis* (Fig. 1): all NPN lacked the duplicated region D', and only contained *tex* from region E, with the pseudo cytosine methyltransferases not identified during blast searches. *N. subflava* was the only NPN species in which the putative regions A, B, C and D were contiguous, although the putative regions A, B and C were contiguous or nearly contiguous in all species, apart from *N. oralis*, *N. animalis, N. canis* and *N. bacilliformis.* In the case of *N. canis*, *N. animalis, N. elongata* subsp. *elongata* and *N. bacilliformis*, the different regions were not located on a single contig, but the separation of these regions being an artefact was rejected based on comparisons to closed genome sequences of these species. The gene encoding *galE* from region D was not present in all NPN found to contain a *cps*, and in *N. animalis*, *N. oralis* and both *N. elongata* isolates, it was found to be near or adjacent to region A, rather than contiguously with the other region D genes as is the case in *N. meningitidis*.

### Homologous region A genes among species

**Fig. 2.**
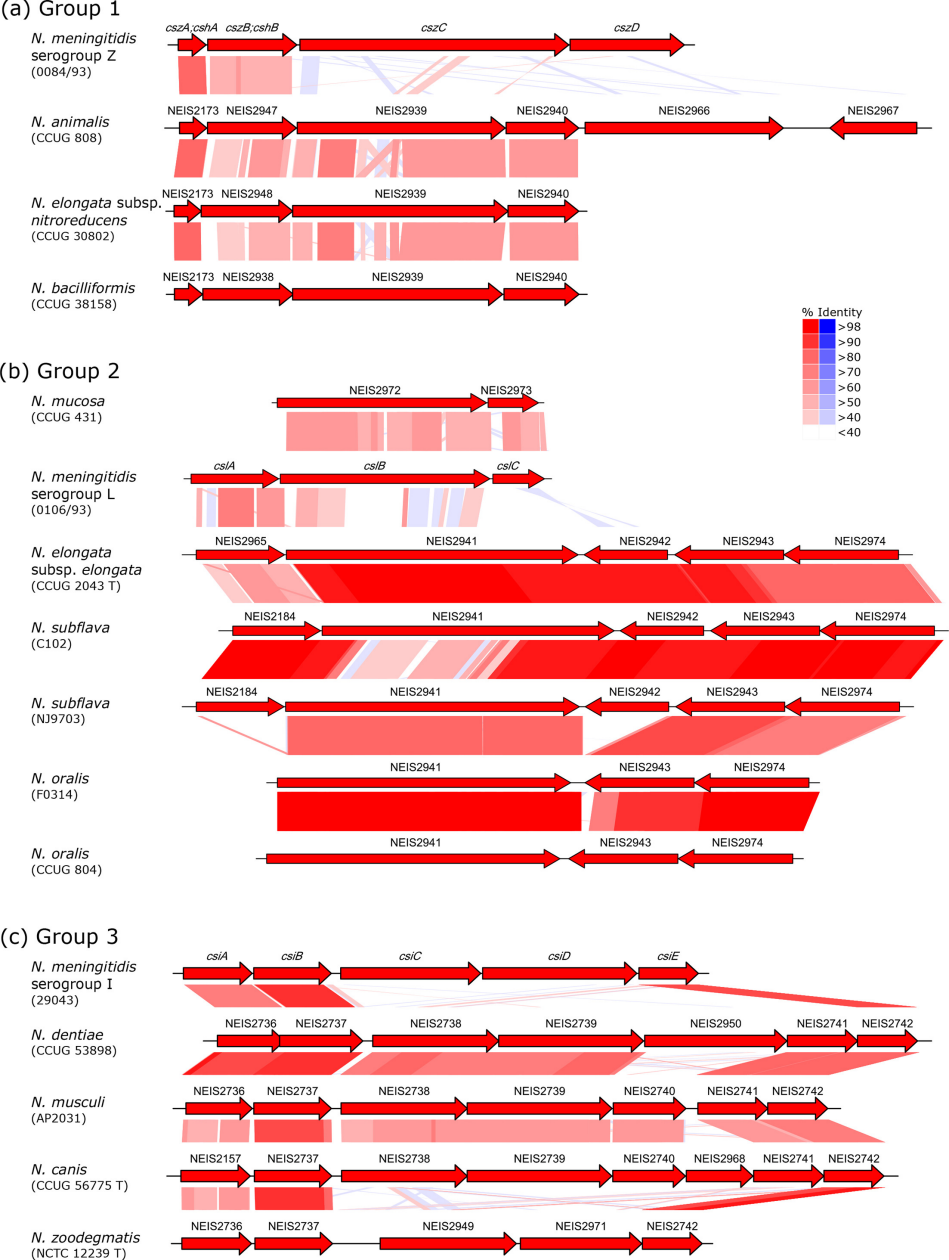
Pairwise tBLASTx comparisons of candidate region A genes between representative *Neisseria* species proposed to contain region A genes. Isolates within each group, 1 (a), 2 (b) or 3 (c) share homologous region A genes. Red and blue indicate shared amino acid identity between isolates, with higher intensity indicating higher sequence similarity. Blue indicates an inversion. Between species variation in nucleotide sequence identity was >97 %; hence, one representative is provided for each species, with three exceptions: two *N. elongata* isolates belong to totally different groups [1 (a)/ 3 (b)]; one of the four *N. oralis* isolates had a variant of NEIS2943 that shared only 81 % nucleotide identity with other *N. oralis* isolates, and two of the six *N. subflava* isolates had a variant of NEIS2941 that shared only 71–73 % nucleotide identity with other *N. subflava* isolates (b).

In some instances, several isolates from each species possessed a *cps: N. bacilliformis* (four isolates); *N. subflava* (six isolates); and *N. oralis* (four isolates). Each gene found in *N. bacilliformis* shared >98 % nucleotide identity with the corresponding gene in all other isolates, indicating that all four isolates shared a highly similar *cps*. In *N. subflava*, four of the five candidate genes shared >97 % nucleotide identity among isolates, although three isolates were missing a predicted acetyltransferase. The identity scores for the other gene, a predicted glycosyltransferase, were either 71–73 or 97–100 %, which indicated that there were two versions of this gene (Fig. 2b). *N. oralis* had three region A candidates, two of which shared >98 % nucleotide identity among isolates. The third only had identity scores of >98 % among three of the four isolates, with the version in CCUG 804 only sharing 81 % identity with the others (Fig. 2b).

Based on similarities in *cps* organization, and the results of blast searches during region A annotation, it also became clear that there was shared region A homology between NPN species. Homologous genes were consistently grouped, such that if a group of species shared one gene, they were likely to share another gene, with three homology groups identified in total (Fig. 2). Pairwise comparisons of amino acid sequences generated by Clustal were used to analyse these groups further.

Group 1 contained putative region As from *N. animalis, N. elongata* subsp. *nitroreducens* and *N. bacilliformis*, which shared four homologous genes with 78–83, 42–47, 56–58 and 61–64 % aa identity between species (Fig. 2a). The first two genes also shared 73–81 and 42–47 % aa identity with *cszA/cshA* and *cszB/cshB*, respectively, which are found at the beginning of the region A of serogroups H and Z *N. meningitidis.*

Group 2 contained putative region A from *N. elongata* subsp. *elongata*, *N. subflava* and *N. oralis*, which shared up to five homologous genes with 58, 60–95 , 97 , 86–95  and 75–>99 % aa identity between species, although *N. oralis* was missing the first and third genes, and three isolates of *N. subflava* lacked the third gene (Fig. 2b). The first two genes also shared 61–72 and 32–33 % aa identity with *cslA* and *cslB*, respectively, which are found at the beginning of the region A of serogroup L *N. meningitidis*, although the *cslB* homologue was 40 % longer. *N mucosa* also possessed a gene with 59 % aa identity with *cslB*, differing in length by only 3 bp, as well as a homologue with 56 % aa identity to *cslC*.

Group 3 contained putative region As from *N. dentiae*, *N. musculi*, *N. canis*, *N. zoodegmatis* and *N. animaloris*, although *N. animaloris* was not annotated further due to its incomplete assembly. These isolates shared up to seven homologous genes with 56–96, 86–94 , 70–82 , 73–85 , 65 , 68–83  and 79–90 % aa identity between species (Fig. 2c). *N. zoodegmatis* only contained the first two and last one of these genes, whilst *N. dentiae* lacked the fifth gene. The first two genes and last gene were also 71–74, 83–89 and 76–86 % homologous to *csiA*, *csiB* and *csiE* from serogroup I *N. meningitidis*, respectively, with the exception of the first gene in *N. canis,* which had only 56 % aa identity with *csiE* and 68 % aa identity with *csaA* from serogroup A *N. meningitidis*.

### Distribution of *cps* homologues among *Neisseria* species

**Fig. 3.**
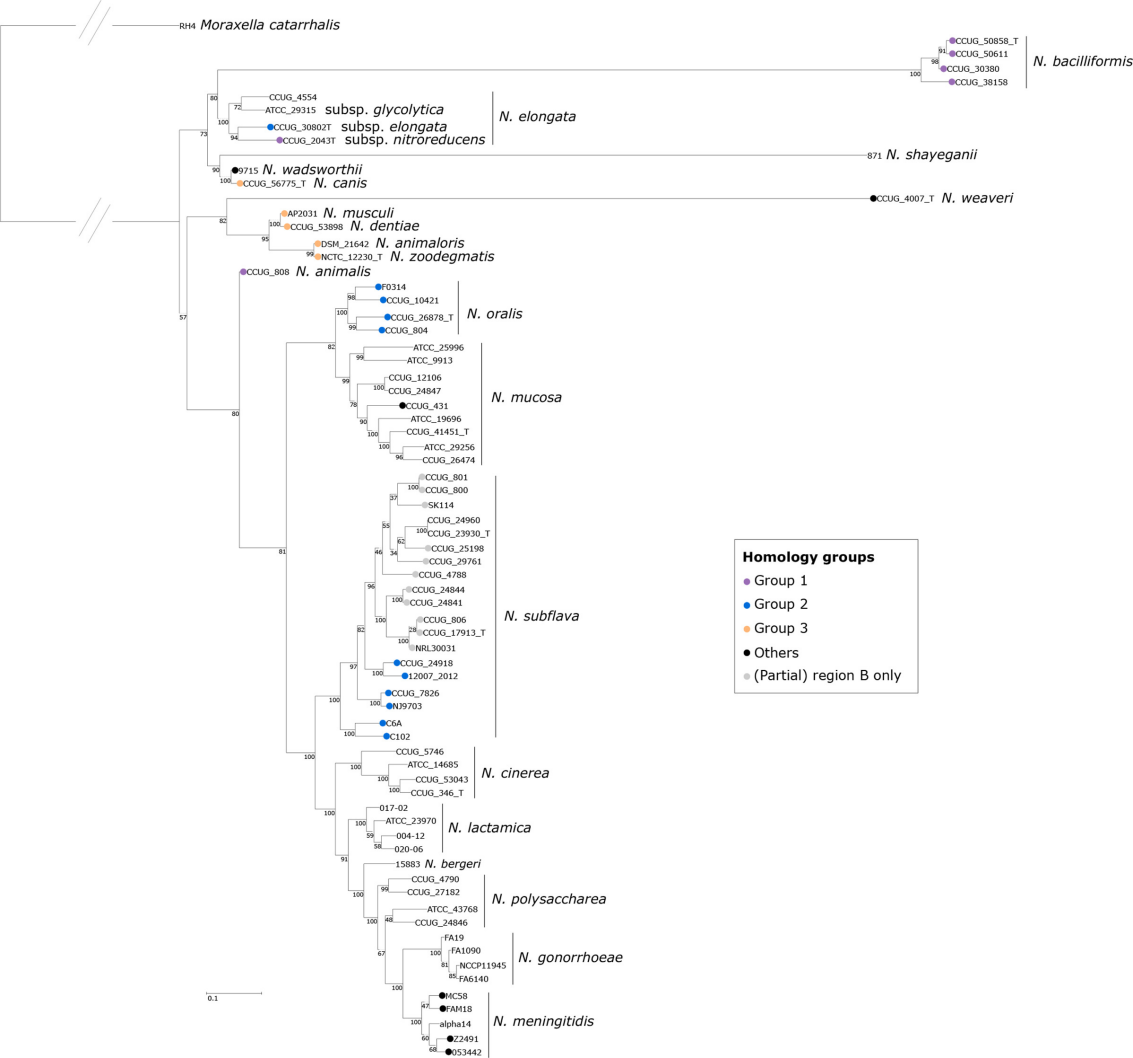
An ML phylogeny generated from an alignment of concatenated rMLST nucleotide sequences from *Neisseria*, with *Moraxella catarrhalis* as an outgroup. Branch supports are based on 100 bootstrap replicates. Corrected for recombination in ClonalFrameML. Coloured circles indicate the presence of capsule genes. Isolates sharing homology in region A, as shown in Fig. 2, are filled with the same colour. Other isolates are black. Grey circles indicate partial region B genes identified only.

Mapping the presence of *cps* onto the phylogeny of *Neisseria* species reconstructed from rMLST [24] sequences indicated that *cps* genes were common and widely distributed among *Neisseria* (Fig. 3). Species sharing homologous region A genes did not necessarily belong to a monophyletic group. *N. cinerea*, *N. lactamica*, *N. polysaccharea* and *N. gonorrhoeae* all belonged to a monophyletic group with *N. meningitidis*, and, with the exception of *N. meningitidis*, no isolates from these species were found to possess a complete *cps* locus. The closest encapsulated relative of *N. meningitidis* was *N. subflava*. Absences of *cps* genes in other species were more sporadic, with 2 of 4 *N. elongata* subsp. isolates, *Neisseria shayeganii* (1 isolate), 9 of 10 *N. mucosa* and 13 of 19 *N. subflava* lacking *cps*, although 11 of the *N. subflava* isolates contained remnants of region B genes.

**Fig. 4.**
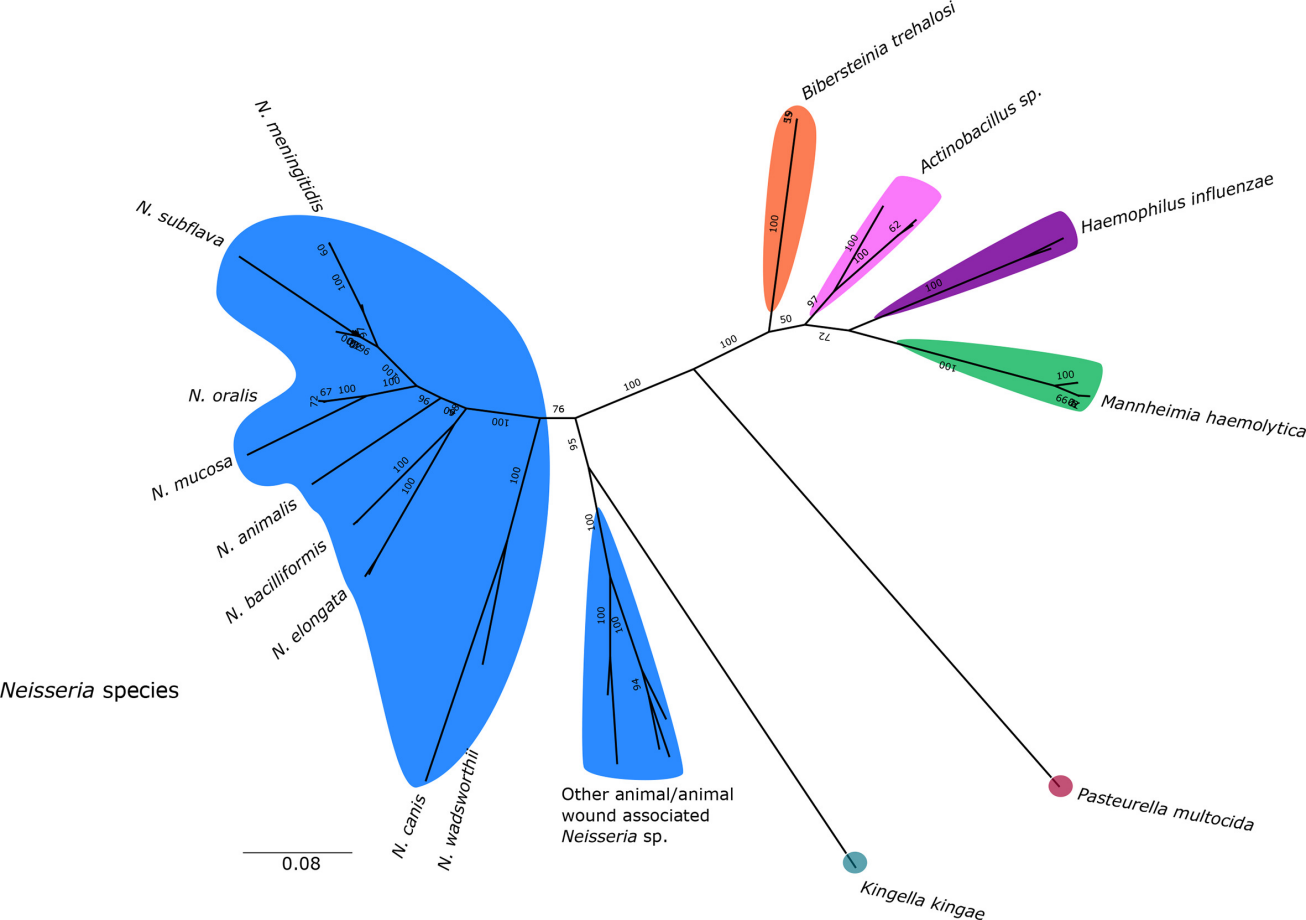
An unrooted ML phylogeny with 100 bootstraps, generated from an alignment of concatenated amino acid sequences of *ctrABCDEF* (or their equivalent homologues) from *Neisseria* species, and other members of the *Neisseriaceae* and *Pasteurellaceae*.

NCBI pBLAST searches indicated that *Kingella kingae*, *B. trehalosi*, *Actinobacillus* sp., *Mannheimia haemolytica* and *H. influenzae* possessed homologues of *N. meningitidis* region B and C genes. An unrooted ML phylogeny generated from aligned amino acid sequences of region B and C homologues in these species, *P. multocida* and *Neisseria* indicated that genes in *N. meningitidis* were more closely related to those from *N. subflava* and most other *Neisseria* than any other genera (Fig. 4).

## Discussion

Among the *Neisseria,* the polysaccharide capsule has been considered to be a virulence factor unique to *N. meningitidis.* Although region B and C genes had been identified previously in an isolate of *N. subflava,* in the absence of further evidence at the time, this was attributed to an isolated HGT event facilitated by a DNA uptake sequence in *ctrA* [21]. In this study, homologues of all the conserved region B and C genes in multiple NPN species from across the genus have been identified, with accompanying putative capsule synthesis loci. On the balance of evidence from genomic data, including the comparable synteny between most NPN species and the meningococcal *cps* (Fig. 1), and the high homology of several putative capsule synthesis genes to those of *N. meningitidis* and other species (Fig. 2, Table 1), the candidate region A genes identified were most likely to function in capsule synthesis. The discovery of capsule genes in non-pathogenic bacteria is not unprecedented, with a similar finding in the *Streptococcus mitis* group streptococci overturning the assumption that capsule production was unique to the pathogenic *Streptococcus pneumoniae* [41]. In common with many virulence factors [42], including the type IV pilus [20, 21], the capsule might be better described as a ‘host adaptation factor’ [21], with effects on pathogenic potential being incidental.

Region A annotations were consistent with the potential for more than one capsular group within *N. elongata*, and possibly *N. subflava* and *N. oralis* (Fig. 2). Differences observed among *N. subflava* isolates were comparable to the divergence between the polysialyltransferase-encoding *csb* and *csc*, which give rise to the structural differences between meningococcal serogroups B and C, respectively [15, 43]. The presence of multiple groups or types is commonplace among Gram-negative bacteria, including *E. coli*, *H. influenzae* and *Mannheimia haemolytica* [4–6]. In *E. coli*, over 80 structurally different capsules exist, some of which are associated with specific pathologies, or are only expressed at certain temperatures [4]. The range of niches exploited by *E. coli*, including different hosts and tissues, as well as free-living environments, may be responsible for this diversity [44]. *Neisseria* do not demonstrate such a wide exploitation of niches, but it has been demonstrated that different species have tropisms for specific nasopharyngeal sites [45]. In *N. meningitidis*, isolates expressing a capsule from serogroups A, B, C, W, X or Y are associated with IMD [2], leading to an interest in the evolutionary history of the capsule.

Models presented previously, hypothesizing that *N. meningitidis* must have acquired a capsule by HGT [3, 22], can be re-examined in light of the data presented here. The identification of capsules in 13 NPN species does not preclude the acquisition of capsule genes in *N. meningitidis* by HGT. Notably, capsule genes have still not been identified in any isolates belonging to the monophyletic group that contains *N. cinerea*, *N. lactamica*, ‘*N. bergeri’*, *N. polysaccharea*, *N. gonorrhoeae* and *N. meningitidis*, with the exception of *N. meningitidis* itself (Fig. 3). This uneven distribution of capsule genes must be explained by acquisition and/or loss [46, 47]. Given the predicted common ancestor of these species, for the capsule to be present only in certain *N. meningitidis* isolates, the capsule genes must either have been lost independently as many as six times, or lost once in a common ancestor of the monophyly and re-acquired in *N. meningitidis.* The latter is the most evolutionarily parsimonious explanation, although a scenario between these two extremes is also possible. Further support for an HGT event comes from the duplication of region D genes within the *N. meningitidis cps*, which is attributed to illegitimate recombination in the *galE* gene in a model proposed by Bartley *et al.* [22]. A satisfactory alternative explanation for the organization of the meningococcal *cps* has not been proposed to date.

It has been posited that the donor of capsule genes to *N. meningitidis* may have been a member of the *Pasteurellaceae*, based on *cps* organization and sequence similarity between regions B and C of *N. meningitidis* and equivalent genes in *P. multocida* [3]. This is not consistent with the phylogenetic data presented here, which show that regions B and C of the *N. meningitidis cps* more closely resembled homologues of *N. subflava* than any other genus (Fig. 4). Therefore, if the *N. meningitidis cps* was acquired by HGT, regions B and C at least were more likely to have been acquired from another *Neisseria* species. Recombination between closely related species is more probable, due to higher similarity of flanking sequences and the increased potential for a compatible DNA uptake sequence. HGT between *Neisseria* species has been described previously, including cross-species exchanges of *pilE,* another gene with links to virulence [21, 48]. Acquisition of capsule genes in *H. influenzae* has also been proposed to be a result of HGT from a commensal species of the same genus [49]. Interestingly, the suggested organization of the donor ancestral *cps* island described in the model by Bartley *et al.* [22] matches the organization of the *N. subflava cps* described in the present study; however, a potential donor could alternatively be a close relative that has either not been previously isolated or since become extinct.

The origin of region A genes in *N. meningitidis*, responsible for the differences in capsule serogroups, is less clear, since none of the isolates annotated here possessed a full complement of region A loci with a close resemblance to *N. meningitidis* serogroups. Based on sequence similarity among capsule synthesis genes of *Haemophilus*, *Actinobacillus*, *Mannheimia* and *Neisseria*, a case for horizontal exchange of capsule synthesis genes across genera and the formation of mosaic complements of genes has been made [50, 51]. The lower G+C content of region A (as low as 25–45 %) compared to the rest of the genome (~50 %), a phenomenon also seen in some *E. coli* capsular types, has also been cited as evidence for cross-genus horizontal acquisition of capsule synthesis genes [15, 52]. The exact nature of this potentially complex evolutionary history, and the degree of exchange in recent evolutionary time, remain unclear.

The discovery of capsule genes in NPN highlights the polysaccharide capsule’s role in asymptomatic colonization and transmission, an important stage in meningococcal epidemiology; however, the acquisition of capsule by some genotypes of *N. meningitidis* has had an important impact on their behaviour, increasing their propensity to cause disease. Sequence similarity between NPN capsule genes and *N. meningitidis* sheds some light on the complicated evolutionary processes in these highly transformable organisms. Further sequencing of NPN, as well as other oral and nasopharyngeal commensals, may provide additional insights into the emergence of pathogenic serogroups in this important pathogen.

## Data bibliography

1. Schoen C, Blom J, Claus H, Schramm-Gluck A, Brandt P *et al*. pubMLST id 30 (2008).
2. Tettelin H, Saunders NJ, Heidelberg J, Jeffries AC, Nelson KE *et al*. pubMLST id 240 (2000).
3. Parkhill J, Achtman M, James KD, Bentley SD, Churcher C *et al*. pubMLST id 613 (2000).
4. Bentley SD, Vernikos GS, Snyder LA, Churcher C, Arrowsmith C *et al*. pubMLST id 698 (2007).
5. Lewis LA, Gillapsy AF, McLaughlin RE, Gipson M, Ducey TF *et al*. pubMLST id2855 (2015).
6. Bennet JS, Jolley KA, Earle SG, Corton C, Bentley SD *et al*. pubMLST ids 2863, 19077, 19091, 49339-49342, 49345-49349, 49351-49353 and 49358-49368 (2012).
7. Marri PR, Paniscus M, Weyand NJ, Rendon MA, Calton CM *et al*. pubMLST ids 3565, 1473903 and 14740 (2010).
8. Bennet JS, Jolley KA, Maiden MC. pubMLST ids 5197, 5354, 8778, 8837, 19940, 20515, 20516, 21038-21043 and 21045-21047 (2013).
9. Fulton L, Clifton S, Chinwalla AT, Mitreva M, Sodergren E *et al*. pubMLST id 5544 (2013).
10. Bennet JS, Bentley SD, Vernikos GS, Quail MA, Cherevach I *et al*. pubMLST id 8851 (2010).
11. Peng J, Yang L, Yang F, Yang J, Yan Y *et al*. pubMLST id 12672 (2008).
12. Chung GT, Yoo JS, Oh HB, Lee YS, Cha SH *et al*. pubMLST id 13685 (2008).
13. HMP Consortium. pubMLST ids 21044, 21048, 21049 and 21060 (2013).
14. Harrison OB, Bennet JS, Derrick JP, Maiden MC, Bayliss CD. pubMLST ids 21063 and 21064; GenBank accession no. HF562984.1 (2013).
15. Irish Meningococcus Genome Library. pubMLST id 26870 (2013).
16. Weyand NJ, Ma M, Phifer-Rixey M, Taku NA, Rendon MA *et al*. pubMLST id 29520 (2016).
17. Wolfgang W. pubMLST id 30325 (2016).
18. Liu G. pubMLST id 36317 (2015).
19. Abrams AJ, Trees DL, Nicholas RA. pubMLST ids 46275 and 46276 (2015).
20. Chong TM, Ng KT, Chan KG. pubMLST id 46753 (2014).
21. Craig Venter Institute. pubMLST id 49373 (2016).
22. Parkhill J. NCTC 3000 samples ERS950465, ERS1058919 and ERS980032 (2017).
23. Pathogen Informatics (Sanger). GenBank accession no. LT571436.1 (2016).
24. Muzny D, Qin X, Deng J, Jiang H, Liu Y *et al*. GenBank accession no. GL878494.1 (2013).
25. Calcutt MJ, Foecking MF, Mhlanga-Mutangadura T, Reilly TJ. GenBank accession no. CP009159.1 (2014).
26. MacInnes J, Kropinski AM, Nash JHE. GenBank accession no. CP003875.1 (2017).
27. May BJ, Zhang Q, Li LL, Paustian ML, Whittam TS *et al*. GenBank accession no. AE004439.1 (2014).
28. Harhay GP, McVey DS, Koren S, Phillippy AM, Bono J *et al*. GenBank accession no. CP006954.1 (2014).
29. Harhay GP, McVey S, Clawson ML, Bono J, Heaton MP *et al*. GenBank accession no. CP006955.1 (2014).
30. Harhay GP, McVey S, Clawson ML, Bono J, Heaton MP *et al*. GenBank accession no. CP006956.1 (2014).
31. Harhay GP, Koren S, Phillippy A, McVey DS, Kuszak J *et al*. GenBank accession no. CP003745.1 (2013).
32. Buettner F, Martinez-Arias R, Goesmann A, Baltes N, Tegetmeyer H *et al*. GenBank accession no. CP001091.1 (2014).
33. Xu Z, Zhou Y, Li L, Zhou R, Xiao S *et al*. GenBank accession no. CP000687.1 (2014).
34. Foote SJ, Bosse JT, Bouevitch AB, Langford PR, Young NM *et al*. GenBank accession no. CP000569.1 (2014).
35. Su Y-C, Horhold F, Singh B, Riesbeck K. GenBank accession no. CP005967.1 (2014).
36. Bidet P. GenBank accession no. LN869922.1 (2015).
37. Harhay GP, McVey S, Clawson ML, Bono J, Heaton MP *et al*. GenBank accession no. CP006957.1 (2015).
38. Hauglund MJ, Tatum FM, Bayles DO, Maheswaran SK, Briggs RE. GenBank accession no. CP006573.1 (2015).
39. Iskander M, Hayden K, Van Domselaar G, Tsang R. GenBank accession no. CP017811.1 (2016)
40. Heaton MP, Harhay GP, Smith TP, Bono JL, Chitko-McKown CG. GenBank accession no. CP011099.1 (2015).
41. Koren S, Harhay GP, Smith TPL, Bono JL, Harhay DM *et al*. GenBank accession no. CP006619.1 (2013).
42. Haugland MJ, Tatum FM, Bayles DO, Chriswell BO, Maheswaran SK *et al*. GenBank accession no. CP005972.1 (2013).
43. Eidam C, Poehlein A, Brenner Michael G, Kadlec K, Liesegang H *et al*. GenBank accession no. CP005383.1 (2015).
44. Harhay GP, Koren S, Phillippy AM, McVey DS, Kuszak J *et al*. GenBank accession no. CP004752.1 (2017).
45. Hauglund MJ, Tatum FM, Bayles DO, Chriswell BO, Maheswaran SK *et al*. GenBank accession no. CP006574.1 (2015).
46. Zomer A, de Vries SP, Riesbeck K, Meinke AL, Hermans PW *et al*. GenBank accession no. AMSO00000000.1 (2014).
47. Jolley KA, Kalmusova, Feil EJ, Gupta S, Musilek M *et al*. pubMLST ids 985 and 1575 (2000).

## Supplementary Data

Supplementary File 1
