# Supplementary material for: Characterization of capsule genes in non-pathogenic Neisseria species
Source: Microb Genom. 2018 Aug 3;4(9):e000208. doi: 10.1099/mgen.0.000208 (PMC6202450; doi:10.1099/mgen.0.000208)
Supplement: Supplementary File 1 [file mgen-4-208-s001.pdf]

Table S1a: Isolates containing *ctrA*

Isolate	Species	% Identity	Mismatches	Gaps	E-value	Query Cover	GC
CCUG 808	*N. animalis*	79.51	75	1	0	0.95	46.68
DSM 21642	*N. animaloris*	55.44	170	2	5e-142	0.99	47.44
CCUG 50858 T	*N. bacilliformis*	76.38	88	2	0	0.97	49.61
CCUG 30380	*N. bacilliformis*	74.74	97	2	0	1	49.61
CCUG 38158	*N. bacilliformis*	74.74	97	2	0	1	49.7
CCUG 50611	*N. bacilliformis*	75	96	2	0	1	49.61
CCUG 56775 T	*N. canis*	69.35	113	1	7e-172	0.95	48.06
CCUG 53898	*N. dentiae*	56.96	163	3	2e-144	0.99	46.68
CCUG 30802T	*N. elongata* subsp. Elongata	73.32	97	3	0	1.03	48.87
CCUG 2043T	*N. elongata* subsp. Nitroreducens	73.52	102	1	0	0.99	48.94
CCUG 431	*N. mucosa*	68.34	117	1	2e-179	0.97	49.87
AP2031	*N. musculi*	58.65	147	3	2e-133	0.95	46.04
CCUG 26878 T	*N. oralis*	76.35	91	1	0	0.99	48.94
F0314	*N. oralis*	76.09	92	1	0	0.99	49.02
CCUG 10421	*N. oralis*	76.35	91	1	0	0.99	49.11
CCUG 804	*N. oralis*	76.35	91	1	0	0.99	48.85
NJ9703	*N. subflava*	76.84	90	1	0	1.01	48.09
C102	*N. subflava*	76.84	90	1	0	1.01	48.26
12007_2012	*N. subflava*	77.1	89	1	0	1.01	48.43
C6A	*N. subflava*	76.84	90	1	0	1.01	48.09
CCUG 7826	*N. subflava*	77.1	89	1	0	1.01	48.26
CCUG 24918	*N. subflava*	76.84	90	1	0	1.01	48.18
9715	*N. wadsworthii*	61.89	148	1	2e-165	1	47.24
CCUG 4007 T	*N. weaveri*	54.76	172	3	6e-140	0.99	47.47

Table S1b: Isolates containing *ctrB*

Isolate	Species	% Identity	Mismatches	Gaps	E-value	Query Cover	GC
CCUG 808	*N. animalis*	77.63	86	1	0	0.97	46.96
DSM 21642	*N. animaloris*	66.4	125	0	8e-164	0.96	45.61
CCUG 50858 T	*N. bacilliformis*	79.11	75	0	0	0.9	48.76
CCUG 30380	*N. bacilliformis*	79.11	75	0	0	0.9	48.76
CCUG 38158	*N. bacilliformis*	78.83	76	0	0	0.9	48.68
CCUG 50611	*N. bacilliformis*	79.11	75	0	0	0.9	48.76
CCUG 56775 T	*N. canis*	73.1	99	0	1e-172	0.95	46.74
CCUG 53898	*N. dentiae*	68.72	117	0	4e-169	0.97	46.35
CCUG 30802T	*N. elongata* subsp. Elongata	74.49	95	1	0	0.98	47.46
CCUG 2043T	*N. elongata* subsp. Nitroreducens	74.23	96	1	0	0.98	47.46
CCUG 431	*N. mucosa*	74.68	96	1	0	1.01	48.17
AP2031	*N. musculi*	69.48	112	0	2e-168	0.92	46.19
CCUG 26878 T	*N. oralis*	78.57	80	2	0	0.98	47.62
F0314	*N. oralis*	78.32	81	2	0	0.98	47.7
CCUG 10421	*N. oralis*	78.57	80	2	0	0.98	47.62
CCUG 804	*N. oralis*	78.83	79	2	0	0.98	47.62
NJ9703	*N. subflava*	76.79	85	2	0	0.98	48.12
C102	*N. subflava*	76.79	85	2	0	0.98	48.21
12007_2012	*N. subflava*	76.53	86	2	0	0.98	48.29
C6A	*N. subflava*	76.79	85	2	0	1.01	48.12
CCUG 7826	*N. subflava*	76.79	85	2	0	1.01	48.12
CCUG 24918	*N. subflava*	76.79	85	2	0	1.01	47.86
9715	*N. wadsworthii*	72.05	102	0	6e-171	0.91	45.83

Isolate	Species	% Identity	Mismatches	Gaps	E-value	Query Cover	GC
CCUG 4007 T	*N. weaveri*	66.84	124	0	2e-162	0.97	45.94

Table S1c: Isolates containing *ctrC*

Isolate	Species	% Identity	Mismatches	Gaps	E-value	Query Cover	GC
CCUG 808	*N. animalis*	82.71	46	0	3e-142	0.97	50.5
DSM 21642	*N. animaloris*	76.32	63	0	4e-139	0.97	52.88
CCUG 50858 T	*N. bacilliformis*	80.08	53	0	2e-147	0.97	55.26
CCUG 30380	*N. bacilliformis*	80.08	53	0	6e-143	0.97	55.26
CCUG 38158	*N. bacilliformis*	80.83	51	0	4e-136	0.97	55.14
CCUG 50611	*N. bacilliformis*	80.08	53	0	7e-143	0.97	55.26
CCUG 56775 T	*N. canis*	75.94	64	0	3e-132	0.97	52.51
CCUG 53898	*N. dentiae*	77.07	61	0	1e-132	0.97	51.38
CCUG 30802T	*N. elongata* subsp. Elongata	82.33	47	0	6e-140	0.97	51.5
CCUG 2043T	*N. elongata* subsp. Nitroreducens	82.33	47	0	4e-140	0.97	51.5
CCUG 431	*N. mucosa*	78.2	58	0	6e-135	0.97	52.51
AP2031	*N. musculi*	75.56	65	0	5e-130	0.97	49.87
CCUG 26878 T	*N. oralis*	82.33	47	0	3e-141	0.97	52.26
F0314	*N. oralis*	82.33	47	0	1e-140	0.97	52.38
CCUG 10421	*N. oralis*	82.33	47	0	1e-140	0.97	52.38
CCUG 804	*N. oralis*	82.33	47	0	1e-140	0.97	52.26
NJ9703	*N. subflava*	79.7	54	0	9e-132	0.97	53.01
C102	*N. subflava*	79.32	55	0	1e-130	0.97	53.26
12007_2012	*N. subflava*	79.32	55	0	9e-131	0.97	53.26
C6A	*N. subflava*	79.7	54	0	3e-131	0.97	52.88
CCUG 7826	*N. subflava*	79.7	54	0	2e-131	0.97	53.01
CCUG 24918	*N. subflava*	80.08	53	0	2e-131	0.97	53.13
9715	*N. wadsworthii*	78.95	56	0	2e-139	0.97	51.5
CCUG 4007 T	*N. weaveri*	78.2	58	0	1e-139	0.97	52.13

Table S1d: Isolates containing *ctrD*

Isolate	Species	% Identity	Mismatches	Gaps	E-value	Query Cover	GC
CCUG 808	*N. animalis*	90.7	20	0	3e-130	0.99	46.7
DSM 21642	*N. animaloris*	89.3	23	0	7e-130	0.99	45.16
CCUG 50858 T	*N. bacilliformis*	87.91	26	0	6e-129	0.99	48.39
CCUG 30380	*N. bacilliformis*	87.91	26	0	1e-126	0.99	48.08
CCUG 38158	*N. bacilliformis*	87.91	26	0	1e-125	0.99	48.39
CCUG 50611	*N. bacilliformis*	87.91	26	0	7e-126	0.99	48.39
CCUG 56775 T	*N. canis*	90.19	21	0	7e-132	0.99	47.77
CCUG 53898	*N. dentiae*	88.32	25	0	2e-128	0.99	47.77
CCUG 30802T	*N. elongata* subsp. Elongata	87.91	26	0	2e-127	0.99	47.81
CCUG 2043T	*N. elongata* subsp. Nitroreducens	87.91	26	0	2e-127	0.99	48.23
CCUG 431	*N. mucosa*	89.72	22	0	4e-131	0.99	49
AP2031	*N. musculi*	84.04	34	0	1e-121	0.98	47.62
CCUG 26878 T	*N. oralis*	93.09	15	0	1e-137	1	48.69
F0314	*N. oralis*	93.09	15	0	5e-137	1	48.69
CCUG 10421	*N. oralis*	93.09	15	0	5e-137	1	48.69
CCUG 804	*N. oralis*	93.09	15	0	5e-137	1	48.69
NJ9703	*N. subflava*	99.08	2	0	1e-144	1	48.08
C102	*N. subflava*	99.08	2	0	1e-144	1	48.39
12007_2012	*N. subflava*	99.08	2	0	1e-144	1	48.39

Isolate	Species	% Identity	Mismatches	Gaps	E-value	Query Cover	GC
C6A	*N. subflava*	98.62	3	0	3e-144	1	48.39
CCUG 7826	*N. subflava*	98.62	3	0	7e-144	1	48.23
CCUG 24918	*N. subflava*	99.08	2	0	1e-144	1	48.08
9715	*N. wadsworthii*	92.52	16	0	5e-135	0.99	48.69
CCUG 4007 T	*N. weaveri*	89.25	23	0	6e-129	0.99	45.78

Table S1e: Isolates containing *ctrE*

Isolate	Species	% Identity	Mismatches	Gaps	E-value	Query Cover	GC
CCUG 808	*N. animalis*	73.07	182	2	0	0.97	52.28
DSM 21642	*N. animaloris*	66.27	216	4	0	0.95	51.92
CCUG 50858 T	*N. bacilliformis*	74.12	177	2	0	1	52.94
CCUG 30380	*N. bacilliformis*	74.4	175	2	0	1	52.89
CCUG 38158	*N. bacilliformis*	73.69	180	2	0	1	53.01
CCUG 50611	*N. bacilliformis*	74.12	177	2	0	1	52.94
CCUG 56775 T	*N. canis*	51.85	302	6	0	0.92	46.67
CCUG 53898	*N. dentiae*	64.49	197	1	0	0.93	50.09
CCUG 30802T	*N. elongata* subsp. Elongata	64.49	240	3	0	1	52.42
CCUG 2043T	*N. elongata* subsp. Nitroreducens	64.91	237	3	0	1	52.32
CCUG 431	*N. mucosa*	81.53	119	1	0	0.93	52.25
AP2031	*N. musculi*	62.34	220	4	0	0.98	50.42
CCUG 26878 T	*N. oralis*	83.1	108	1	0	0.92	52.35
F0314	*N. oralis*	82.95	109	1	0	0.92	52.2
CCUG 10421	*N. oralis*	83.41	106	1	0	0.92	52.2
CCUG 804	*N. oralis*	83.1	108	1	0	0.92	52.35
NRL30031	*N. subflava*	96.45	25	0	0	1	
NJ9703	*N. subflava*	96.74	23	0	0	1	53.1
C102	*N. subflava*	93.71	39	1	0	0.92	53.04
CCUG 24841	*N. subflava*	96.61	23	0	0	0.96	
CCUG 24844	*N. subflava*	97.59	17	0	0	1	
12007_2012	*N. subflava*	95.18	34	0	0	1	53.19
C6A	*N. subflava*	95.74	30	0	0	1	53.43
CCUG 4788	*N. subflava*	96	15	0	0	0.53	
CCUG 29761	*N. subflava*	95.55	21	0	0	0.67	
CCUG 17913 T	*N. subflava*	96.45	25	0	0	1	
CCUG 806	*N. subflava*	96.45	25	0	0	1	
CCUG 7826	*N. subflava*	96.74	23	0	0	1	53.1
CCUG 24918	*N. subflava*	96.45	25	0	0	1	53
CCUG 800	*N. subflava*	92.47	11	0	6e-88	0.21	
CCUG 801	*N. subflava*	92.47	11	0	6e-88	0.21	
CCUG 25198	*N. subflava*	93.89	14	0	0	0.32	
SK114	*N. subflava*	90.64	32	2	0	0.58	
9715	*N. wadsworthii*	62.57	255	1	0	0.97	48.83
CCUG 4007 T	*N. weaveri*	67.06	223	1	0	0.96	51.23

Table S1f: Isolates containing *ctrF*

Isolate	Species	% Identity	Mismatches	Gaps	E-value	Query Cover	GC
CCUG 808	*N. animalis*	66.42	136	0	0	0.96	55.96
DSM 21642	*N. animaloris*	63.73	147	1	5e-179	0.97	53.53
CCUG 50858 T	*N. bacilliformis*	60.39	139	2	7e-156	0.85	19077
CCUG 30380	*N. bacilliformis*	59.31	161	3	7e-158	0.96	58.15
CCUG 38158	*N. bacilliformis*	59.48	154	2	7e-153	0.92	58.31
CCUG 50611	*N. bacilliformis*	60	152	2	2e-153	0.92	58.23

Isolate	Species	% Identity	Mismatches	Gaps	E-value	Query Cover	GC
CCUG 56775 T	*N. canis*	59.56	163	2	1e-162	0.97	51.66
CCUG 53898	*N. dentiae*	60.14	164	2	9e-171	1	54.57
CCUG 30802T	*N. elongata* subsp. Elongata	60.75	156	1	9e-165	0.95	56.1
CCUG 2043T	*N. elongata* subsp. Nitroreducens	60.75	156	1	1e-163	0.95	56.02
CCUG 431	*N. mucosa*	66.91	133	2	0	0.97	54.8
AP2031	*N. musculi*	60.57	165	1	9e-173	1	54.63
CCUG 26878 T	*N. oralis*	66.58	134	1	0	0.96	54.65
F0314	*N. oralis*	66.34	135	1	0	0.96	54.8
CCUG 10421	*N. oralis*	66.34	135	1	0	0.96	54.58
CCUG 804	*N. oralis*	66.34	135	1	0	0.96	54.73
NRL30031	*N. subflava*	97.62	10	0	0	1	
NJ9703	*N. subflava*	85.57	43	0	3e-168	0.71	55.04
C102	*N. subflava*	91.93	33	0	0	0.97	55.4
CCUG 24841	*N. subflava*	96.9	13	0	0	1	
CCUG 24844	*N. subflava*	97.62	10	0	0	1	
12007_2012	*N. subflava*	94.29	24	0	0	1	54.92
C6A	*N. subflava*	92.18	32	0	0	0.97	55.32
CCUG 4788	*N. subflava*	94.67	4	0	1e-39	0.18	
CCUG 29761	*N. subflava*	71.05	15	2	1e-39	0.27	
CCUG 17913 T	*N. subflava*	97.62	10	0	0	1	
CCUG 806	*N. subflava*	97.62	10	0	0	1	
CCUG 7826	*N. subflava*	93.1	29	0	0	1	55.08
CCUG 24918	*N. subflava*	93.15	28	0	0	0.97	55.08
CCUG 800	*N. subflava*	84.08	32	0	3e-114	0.48	
CCUG 801	*N. subflava*	84.08	32	0	3e-114	0.48	
CCUG 25198	*N. subflava*	96.54	12	0	0	0.83	
SK114	*N. subflava*	94.67	4	0	4e-40	0.18	
9715	*N. wadsworthii*	60.29	160	2	4e-164	0.97	51.58
CCUG 4007 T	*N. weaveri*	63.48	148	1	2e-175	0.97	51.88
